# Macewen’s Sign in Diagnosticating Changes in Intracranial Pressure

**Published:** 1915-11

**Authors:** 


**Macewen's Sign in Diagnosticating Changes in Intracranial Pressure.** Wilcox, Archives of Pediatrics, May, 1915, Ther. Gaz. Oct. 15. Macewen's sign was best determined by placing the stethoscope on the forehead just above the base of the nose The skull was then tapped with the percussing finger or the hammer, over the parietal region, beginning just over the parietal boss, from which the percussing finger should approach to the point at which the stethoscope was applied. This should be carried out on both sides of the head. A typical sign observed in this way consisted in a high-pitched, sharp, short, cracked-pot note. It was most distinct when percussion was being done over, behind, or below the parital boss on either side, was unchanged as the point percussed passed downward, and diminished in intensity and character as the point percussed approached the stethoscope. The latter point was important in differentiating between a false and a true Macewen's sign, as the reverse obtained in percussing a normal skull.

The writer concluded as follows:

(1) The skulls of children of various ages and developments have percussion notes peculiar to the state of the cranium. (2) It is possible to establish a note normal to the various types of crania found in infants and children. (3) A positive Macewen's sign existed when variation from the normal note was found. It consisted in a relative change rather than a definite condition common to all diseased crania. (4) The sign was better elicited by the stethoscope than by the unaided ear. (5) Increased clearness of sound when percussion was done over the posterior portion of the skull rather than near the stethoscope was diagnostic. (6) The sign uniformly accompanied conditions of increased intracranial tension and was not found unless this causative factor existed. (7) It is equally applicable to infants and to older children. (8) It was present in 50 of 53 cases of tuberculous meningitis. (9) It was present in 17 of 18 cases of meningitis of other types.

(10) It was found to vary directly with the development and recession of cerebral symptoms as complications of disease not directly affecting the central nervous system. (11) It was present in 11 of 15 cases of pneumonia, in five of which lumbar puncture showed increased cerebro-spinal fluid under pressure. (12) The sign was uniformly lacking in children normal as to the brain and its coverings.

